# A nomogram to identify appropriate candidates for breast-conserving surgery among young women with breast cancer: A large cohort study

**DOI:** 10.3389/fonc.2022.1012689

**Published:** 2022-10-21

**Authors:** Shengyu Pu, Shaoran Song, Heyan Chen, Can Zhou, Huimin Zhang, Ke Wang, Jianjun He, Jian Zhang

**Affiliations:** ^1^ Department of Breast Surgery, The First Affiliated Hospital of Xi’an Jiaotong University, Xi’an Shaan’xi, China; ^2^ Center for Translational Medicine, the First Affiliated Hospital of Xi’an Jiaotong University, Xi’an Shaan’xi, China

**Keywords:** young breast cancer, breast-conserving surgery, mastectomy, survival, nomogram

## Abstract

**Background:**

There is a gradual increase of female breast cancer under 35 years old, who was characterized as poor prognosis. Whether young patients could obtain greater survival benefits from breast-conserving surgery (BCS) than mastectomy remains controversial.

**Methods:**

Breast cancer patients (≤35 years old) were selected from the Surveillance, Epidemiology, and End Results (SEER) database and divided into BCS and mastectomy group. Propensity score matching (PSM) was used to eliminate the distributional imbalance of variables among two groups. The influence of BCS on overall survival (OS) and breast cancer-specific survival (BCSS) was evaluated by Cox regression. Logistic regression was used to identify factors related to the benefit of BCS and to construct a nomogram. The nomogram was validated by the First Affiliated Hospital of Xi’an Jiaotong University cohort.

**Results:**

Totally, 15,317 cases in the SEER database and 149 cases of external validation cohort were included. BCS was an independent protective factor for OS (*P* = 0.028) and BCSS (*P* = 0.042). A nomogram was established, and the AUC values both in the internal and external validation set were 0.780. The applicability of the model was verified in the PSM cohort and indicated that the survival advantage in the BCS-Benefit group was higher than that in the BCS-Nonbenefit and mastectomy group (*P <*0.001).

**Conclusions:**

For young breast cancer patients, BCS may bring better OS and BCSS than mastectomy, but not all benefit from it. We constructed a model for young patients (≤35 years old) that could identify appropriate candidates who benefit from BCS.

## Introduction

The incidence of breast cancer in young women has been increasing since the mid-1990s and has become a leading cause of cancer death in them (1).There is no consensus on a cutoff age value for defining young women with breast cancer by Eastern and Western scholars. The European Society for Medical Oncology (ESMO) used <40 years old as cutoff age (2), while Chinese researchers regard 35 years old as a reasonable cutoff age. In addition, considering that there is a significant incidence age difference of breast cancer in the worldwide: the average age of breast cancer diagnosis is 45–55 years in China (3), which is 10 years younger than that in Western countries. Therefore, we choose the patients <=35 years old for analysis. Many studies have shown that age <=35 years old was an independent risk factor for local recurrence of breast cancer (4, 5). A previous cohort study reported that the overall survival (OS) and the breast cancer-specific survival (BCSS) rates of patients aged 30 and 30-39 years old were significantly lower than those who were 40-49 or 50-59 (6). The reasons for the poor prognosis in young women with breast cancer are complex, the most important being the more aggressive nature of it, including a high proportion of triple-negative, human epidermal growth factor receptor 2 (HER2) overexpression, grade 3, lymphovascular invasion, and lymphocytic infiltration (7).

The option for local surgical treatment has a significant impact on the prognosis of breast cancer patients. The NSABP B-06 demonstrated that survival outcomes after breast-conserving surgery (BCS) combined with radiotherapy (BCT) were equivalent to those after mastectomy for those with early breast cancer (8). Moreover, A large cohort study found that BCT improved 10-year OS compared with mastectomy (9). However, whether young patients obtain a greater survival benefit from BCS than mastectomy remains controversial. Some analyses of outcomes in young patients who underwent BCS versus mastectomy showed no significant differences in the risk of mortality (10–13). Moreover, some studies have reported that those younger than 35 had an independent risk factor for local recurrence after undergoing BCS (5, 8, 14–17). More recently, several studies have found that patients who underwent BCT have a survival benefit compared to those receiving a mastectomy (9, 18–20).To our knowledge, there are no studies to determine who is more likely to benefit from BCS.

This study aimed to determine who benefits from BCS by extracting breast cancer patients under the age of 35 from the Surveillance, Epidemiology, and End Results (SEER) database for retrospective analysis. Logistic regression was used to screen out factors related to the benefit of BCS and constructed a nomogram. In addition, a cohort from the First Affiliated Hospital of Xi’an Jiaotong University was used for confirmation of the findings. Finally, suitable candidates for BCS were identified and referred for clinical treatment.

## Materials and methods

### Study population and data collection

**Figure 1:**
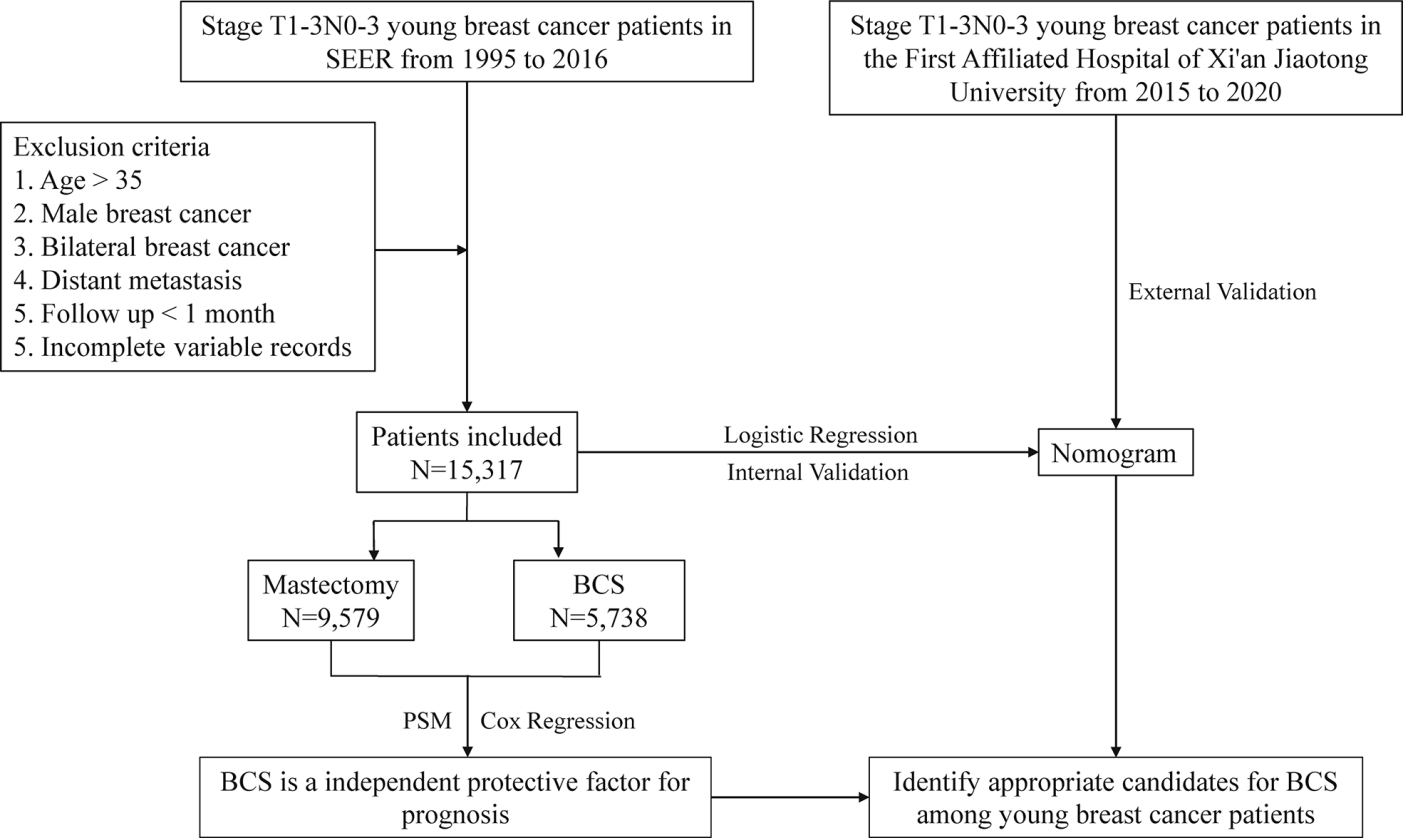
Flow diagram of data analysis.

**Figure 1** shows the process of case screening and analysis. We obtained the data of young female patients with stage T1-3 breast cancer from 1995 to 2016 in the SEER database. The included data were demographic characteristics (age, race, and marital status), tumor-related characteristics (laterality of tumor, grade, histological type, TNM stage, surgical approach, radiation, chemotherapy, and molecular subtype), and follow-up information (survival time and status). Cases with the following characteristics were excluded (1): age > 35 years old (2); male breast cancer (3); bilateral breast cancer (4); distant metastasis (5); follow-up time less than one month (6); incomplete case information. We included 15,317 cases from the SEER database for retrospective analysis. In addition, we also extracted 149 young breast cancer cases who met the inclusion criteria from the First Affiliated Hospital of Xi’an Jiaotong University from 2015 to 2020 as an external validation cohort. The endpoints of this study were OS and BCSS. No intervention or treatment is conducted to patients and the data from SEER database is publicly available, so informed consent is waived in this study.

### Evaluation of the independent protective effect of BCS on prognosis

We divided the samples into two groups according to the surgical approach: the BCS group and the mastectomy group. To adjust for unbalanced variable distributions between the two groups, we performed propensity score matching (PSM) (21) for age, race, marital status, laterality, grade, histology type, AJCC T stage, N stage, radiation, chemotherapy, and subtype. Patients who received a BCS were matched 1:1 on propensity scores with those who received a mastectomy. The standardized mean difference (SMD) was used to evaluate the difference in distribution between the groups for each variable (22). SMD <10% indicated no significant difference. We next observed the differences in OS and BCSS between the two groups before and after PSM using the Kaplan-Meier (KM) survival analysis. Among the PSM cohort, we performed univariate and multivariate Cox regression analyses to evaluate the independent protective effect of BCS on OS and BCSS.

### Construction and validation of a screening nomogram

Entire cohort were randomly divided into training set and validation set in a ratio of 7:3. We performed univariate and multivariate Logistic regression analysis to screen independent predictors of the benefit of BCS in the training set, with a threshold of *P <*0.05.* A* nomogram was then constructed based on the results to quantify the likelihood of a benefit from BCS in young patients and to screen possible candidates for receiving it. Next, we validated the predictive performance of the model on the validation set and external cohort. The discrimination and calibration of the model were evaluated by the time-dependent area under the receiver operating characteristic (ROC) curve (AUC) and calibration curve, respectively. Concurrently, we generated decision curve analysis (DCA) to assess the clinical utility of the model (23). In addition, using the 50% likelihood of benefit based on the score of each patient calculated by the nomogram, we divided the patients in the BCS cohort into two groups: the BCS-Benefit group (benefit possibility>50%) and the BCS-Nonbenefit one (benefit possibility <=50%). The KM survival analysis was performed to compare the OS of patients in the BCS-Benefit, BCS-Nonbenefit, and mastectomy groups to determine if the model could quantify the benefit probability of BCS and identify candidates for receiving it.

### Statistical analysis

The demographic and clinicopathological characteristics were compared using Pearson’s chi-square test. BCSS and OS were observed by Kaplan-Meier analysis and Cox regression analysis, and the survival outcomes were compared using the log-rank test. Logistic regression analysis was used to screen out independent predictors of the benefit of BCS. All statistical analyses were performed with R software (version 4.1.1, R Foundation for Statistical Computing, Vienna, Austria). A two-tailed *P <*0.05 was considered statistically significant.

## Results

### Demographic and clinicopathological features of the patients

**Figure 2:**
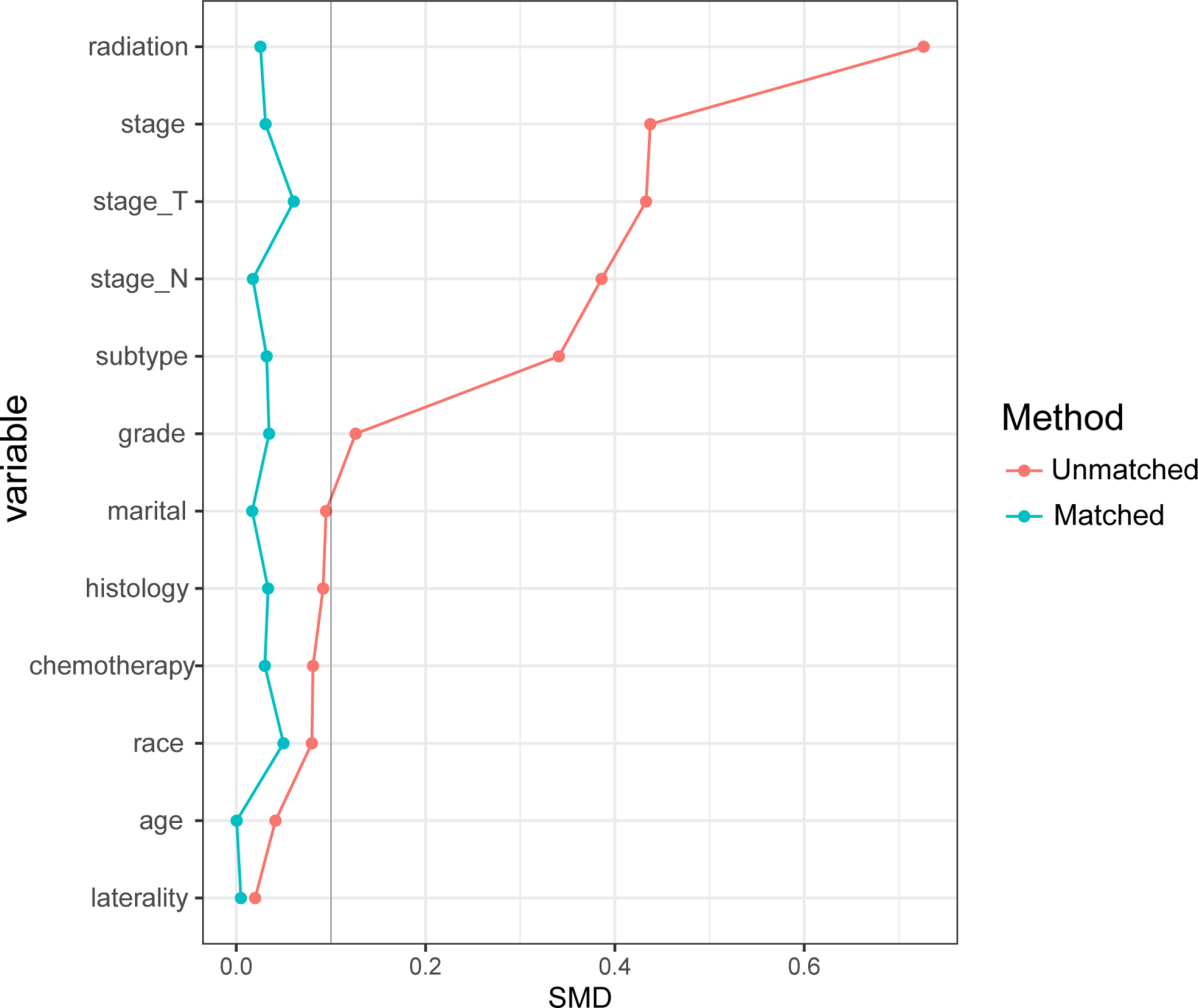
The matching effects of the propensity score matching (PSM).

We included 15,317 cases from the SEER database (**Table 1**) and 149 cases from the First Affiliated Hospital of Xi’an Jiaotong University (**Supplementary Table 1**) for this analysis. As shown in **Table 1**, 5,738 (37.5%) patients received BCS and 9,579 (62.5%) patients received mastectomy. Most patients were white with IDC histology. Patients with low T stage, N stage, and that had received radiation and chemotherapy accounted for the majority. Most variables before PSM were distributed differently between the two groups (SMD >10%). The unbalance distribution was adjusted for all covariates after PSM, and the 3,625 patients that had BCS were matched with 3625 who had a mastectomy. As shown in **Figure 2**, all demographic and clinicopathological characteristics, including age, race, marital status, laterality, histology type, grade, T stage, N stage, radiation, chemotherapy, and molecular subtype, were all balanced between the two groups (SMD <10%).

**Table 1:** Clinical and pathological characteristics for breast cancer patients before and after PSM.

	Unmatched			PSM		
Variables	Non-BCS (%)	BCS (%)	*SMD*	Non-BCS (%)	BCS (%)	*SMD*
	N=9579 (62.5)	N=5738 (37.5)		N=3625 (50.0)	N=3625 (50.0)	
**Age** (Mean (SD))	31.72 (3.12)	31.84 (3.01)	0.041	31.75 (3.11)	31.75 (3.03)	<0.001
**Race (%)**			0.080			0.050
Black	1397 (14.6)	955 (16.6)		607 (16.7)	547 (15.1)	
Other	1108 (11.6)	751 (13.1)		443 (12.2)	477 (13.2)	
White	7074 (73.8)	4032 (70.3)		2575 (71)	2601 (71.8)	
**Marital (%)**			0.095			0.017
No	3720 (38.8)	2496 (43.5)		1450 (40)	1480 (40.8)	
Yes	5859 (61.2)	3242 (56.5)		2175 (60)	2145 (59.2)	
**Laterality (%)**			0.020			0.005
Left	4805 (50.2)	2821 (49.2)		1781 (49.1)	1790 (49.4)	
Right	4774 (49.8)	2917 (50.8)		1844 (50.9)	1835 (50.6)	
**Grade (%)**			0.126			0.035
I	528 (5.5)	480 (8.4)		220 (6.1)	213 (5.9)	
II	3029 (31.6)	1646 (28.7)		1027 (28.3)	1084 (29.9)	
III	5860 (61.2)	3487 (60.8)		2308 (63.7)	2259 (62.3)	
IV	162 (1.7)	125 (2.2)		70 (1.9)	69 (1.9)	
**Histology (%)**			0.092			0.034
IDC	8550 (89.3)	5139 (89.6)		3267 (90.1)	3267 (90.1)	
ILC	181 (1.9)	49 (0.9)		54 (1.5)	41 (1.1)	
Other	848 (8.9)	550 (9.6)		304 (8.4)	317 (8.7)	
**T stage (%)**			0.433			0.061
T1	3739 (39.0)	2996 (52.2)		1605 (44.3)	1590 (43.9)	
T2	4373 (45.7)	2527 (44.0)		1754 (48.4)	1820 (50.2)	
T3	1467 (15.3)	215 (3.7)		266 (7.3)	215 (5.9)	
**N stage (%)**			0.386			0.018
N0	4260 (44.5)	3533 (61.6)		1649 (45.5)	1678 (46.3)	
N1	3506 (36.6)	1692 (29.5)		1461 (40.3)	1434 (39.6)	
N2	1160 (12.1)	358 (6.2)		356 (9.8)	358 (9.9)	
N3	653 (6.8)	155 (2.7)		159 (4.4)	155 (4.3)	
**Chemotherapy (%)**			0.081			0.030
No	1628 (17.0)	1156 (20.1)		628 (17.3)	670 (18.5)	
Yes	7951 (83.0)	4582 (79.9)		2997 (82.7)	2955 (81.5)	
**Radiation (%)**			0.726			0.026
No	5946 (62.1)	1613 (28.1)		1567 (43.2)	1613 (44.5)	
Yes	3633 (37.9)	4125 (71.9)		2058 (56.8)	2012 (55.5)	
**Subtype (%)**			0.341			0.032
HR-/HER2-	879 (9.2)	352 (6.1)		281 (7.8)	288 (7.9)	
HR-/HER2+	283 (3.0)	90 (1.6)		90 (2.5)	78 (2.2)	
HR+HER2-	2222 (23.2)	861 (15.0)		664 (18.3)	635 (17.5)	
HR+/HER2+	803 (8.4)	286 (5.0)		241 (6.6)	239 (6.6)	
Not 2010+	5392 (56.3)	4149 (72.3)		2349 (64.8)	2385 (65.8)	

BCS, Breast conserving surgery; HR, Hormone receptor; IDC, Invasive ductal carcinoma; ILC, Invasive lobular carcinoma; HER2, Human epidermal growth factor receptor 2; PSM, propensity score matched; SMD, Standardized mean differences.

### Influence of BCS on the prognosis among PSM cohort

**Figure 3:**
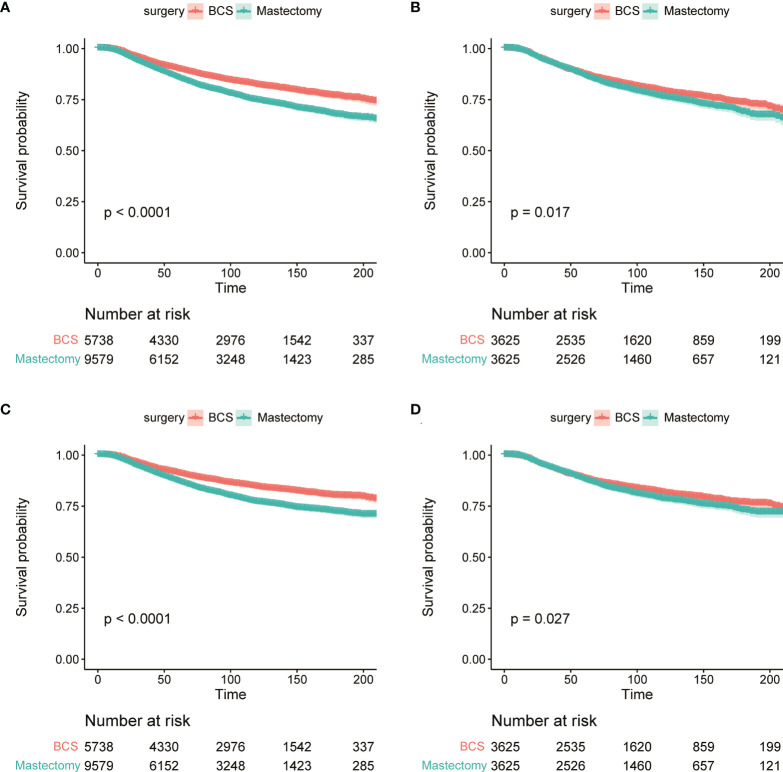
The KM survival analysis of OS **(A, B)** and BCSS **(C, D)** between BCS group and mastectomy group before **(A, C)** and after PSM **(B, D)**.

The OS and BCSS before and after PSM of young breast cancer patients are shown in **Figure 3**. The results revealed that those receiving BCS had a better OS (**Figures 3A, B**). Similarly, those receiving BCS also produced beneficial outcomes for BCSS (**Figures 3C, D**). The detailed 3-year, 5-year, and 10-year OS and BCSS rates are shown in **Table 2**. We also determined the effect of receiving BCS on the prognosis of young breast cancer patients and performed univariate and multivariate Cox regression analysis on OS (**Table 3**) and BCSS (**Supplementary Table 2**). The regression analyses indicated that receiving BCS was a significantly protective factor for OS (mastectomy vs. BCS; HR = 1.127, 95% confidence interval (CI): 1.013–1.254, *P* = 0.028) and BCSS (mastectomy vs. BCS, HR = 1.126; 95% CI: 1.004–1.263, *P* = 0.042). Moreover, other variables such as age, race, grade, T stage, N stage, and molecular subtype were also independent prognostic factors in young breast cancer patients. However, radiation and chemotherapy were not independent factors for OS and BCSS.

**Table 2:** Comparison of patient survival rates between the two surgery groups before and after PSM.

	Before PSM	After PSM
	BCS vs. Mastectomy (95% CI)	BCS vs. Mastectomy (95% CI)
3-year OS rate	0.944 (0.938-0.950) vs.0.921 (0.916-0.927)	0.929 (0.920-0.938) vs. 0.928 (0.919-0.937)
5-year OS rate	0.899 (0.891-0.908) vs. 0.857 (0.849-0.865)	0.873 (0.861-0.885) vs. 0.867 (0.855-0.879)
10-year OS rate	0.821 (0.810-0.833) vs. 0.745 (0.734-0.757)	0.788 (0.772-0.804) vs. 0.762 (0.745-0.779)
3-year BCSS rate	0.950 (0.944-0.956) vs. 0.929 (0.924-0.935)	0.935 (0.927-0.944) vs. 0.936 (0.928-0.944)
5-year BCSS rate	0.911 (0.903-0.919) vs. 0.871 (0.864-0.879)	0.885 (0.874-0.897) vs. 0.879 (0.868-0.891)
10-year BCSS rate	0.844 (0.833-0.855) vs. 0.769 (0.758-0.780)	0.813 (0.798-0.829) vs. 0.784 (0.768-0.801)

BCS, Breast conserving surgery; PSM, propensity score matched; OS, overall survival; BCSS, breast cancer specific survival.

**Table 3:** Univariate and multivariable analysis of overall survival (OS) predictors in breast cancer patients after PSM.

	Univariate analysis			Multivariate analysis		
Variables	HR*	95%CI	*P-*value	HR*	95%CI	*P-*value
**Age**	0.984	(0.967, 1.001)	0.058	0.986	(0.969, 1.003)	0.111
**Race**						
Black	Reference			Reference		
Other	0.593	(0.486, 0.723)	0.000	0.720	(0.589, 0.881)	0.001
White	0.624	(0.548, 0.711)	0.000	0.693	(0.605, 0.792)	0.000
**Laterality**						
Left	Reference					
Right	1.031	(0.927, 1.146)	0.575			
**Marital**						
No	Reference			Reference		
Yes	0.876	(0.787, 0.976)	0.016	0.931	(0.832, 1.042)	0.215
**Grade**						
I	Reference			Reference		
II	2.351	(1.617, 3.417)	0.000	1.958	(1.345, 2.851)	0.000
III	3.373	(2.346, 4.851)	0.000	2.472	(1.711, 3.570)	0.000
IV	3.110	(1.903, 5.082)	0.000	2.546	(1.554, 4.171)	0.000
**Histology**						
IDC	Reference					
ILC	1.036	(0.659, 1.63)	0.879			
Other	0.775	(0.633, 0.95)	0.014			
**T stage**						
T1	Reference			Reference		
T2	1.635	(1.458, 1.834)	0.000	1.294	(1.149, 1.458)	0.000
T3	2.339	(1.925, 2.843)	0.000	1.761	(1.441, 2.154)	0.000
**N stage**						
N0	Reference			Reference		
N1	1.727	(1.519, 1.962)	0.000	1.701	(1.485, 1.948)	0.000
N2	2.750	(2.339, 3.233)	0.000	2.564	(2.157, 3.049)	0.000
N3	4.905	(4.073, 5.908)	0.000	4.342	(3.566, 5.287)	0.000
**Chemotherapy**						
No	Reference			Reference		
Yes	1.392	(1.197, 1.619)	0.000	0.955	(0.809, 1.127)	0.584
**Radiation**						
No	Reference			Reference		
Yes	1.380	(1.236, 1.54)	0.000	0.978	(0.862, 1.110)	0.728
**Surgery**						
BCS	Reference			Reference		
Mastectomy	1.138	(1.023, 1.265)	0.018	1.127	(1.013, 1.254)	0.028
**Subtype**						
HR-/HER2-	Reference			Reference		
HR-/HER2+	0.524	(0.285, 0.964)	0.038	0.545	(0.296, 1.003)	0.051
HR+/HER2-	0.418	(0.305, 0.574)	0.000	0.475	(0.345, 0.654)	0.000
HR+/HER2+	0.279	(0.169, 0.461)	0.000	0.301	(0.182, 0.498)	0.000
Not 2010+	0.69	(0.544, 0.874)	0.002	0.704	(0.553, 0.897)	0.004

BCS, Breast conserving surgery; IDC, Invasive ductal carcinoma; ILC, Invasive lobular carcinoma; HR, Hormone receptor; HER2, Human epidermal growth factor receptor 2; HR*, hazard ratio; CI, confidence interval.

### A nomogram to quantify the benefits of BCS

**Figure 4:**
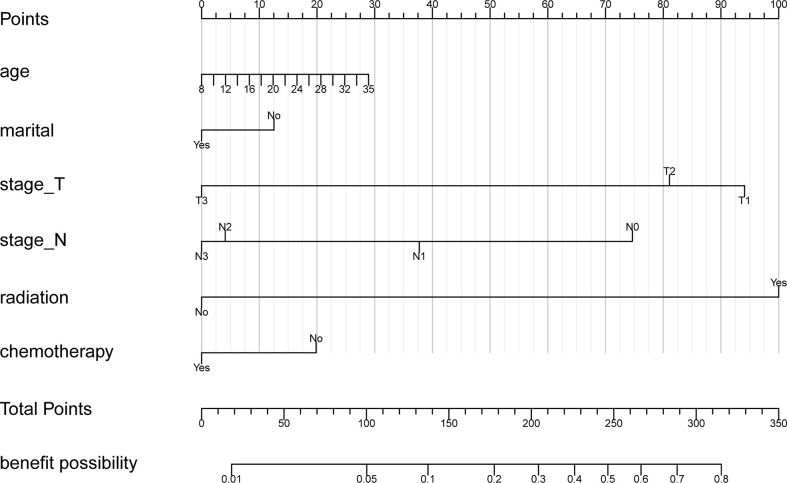
The nomogram to predict the benefit from breast conserving surgery (BCS).

**Figure 5:**
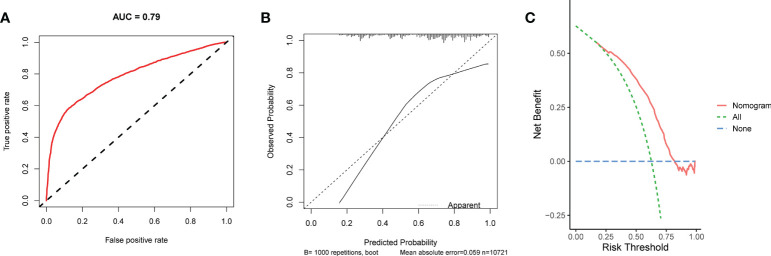
The ROC curve **(A)**, calibration curve **(B)**, and DCA curve **(C)** of the nomogram in the training set.

**Figure 6:**
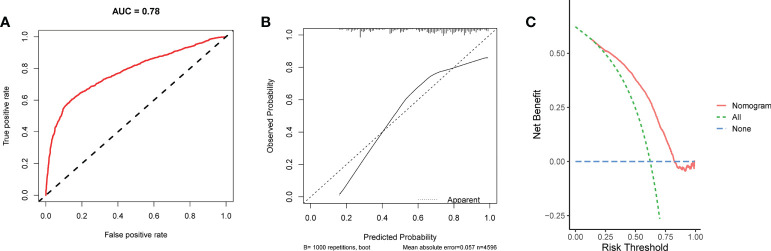
The ROC curve **(A)**, calibration curve **(B)**, and DCA curve **(C)** of the nomogram in the internal validation set.

**Figure 7:**
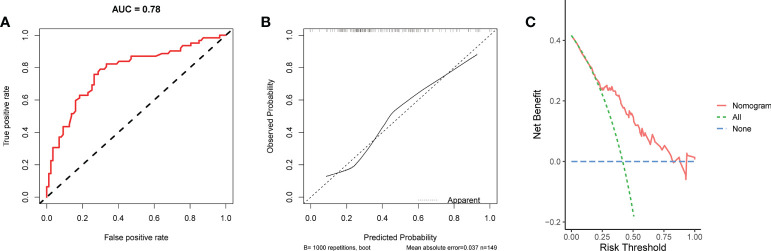
The ROC curve **(A)**, calibration curve **(B)**, and DCA curve **(C)** of the nomogram in the external validation set.

We conducted univariate and multivariate Logistic regression analysis to identify independent factors influencing the benefit of BCS in young breast cancer patients. The age (*P* = 0.002), marital status (*P* < 0.001), T stage (*P* < 0.001), N stage *(P <* 0.001), radiation (*P* < 0.001), and chemotherapy (*P* < 0.001) were screened out as independent influencing factors (**Table 4**). Based on these variables, we established a nomogram to identify candidates for BCS in young patients with T1-3 and N0-3 breast cancer (**Figure 4**). The probability of benefit from BCS was calculated according to the total points in the nomogram (**Supplementary Tables 3** and **4**). ROC and calibration curves were generated to evaluate the discrimination and calibration. The AUC values in the training and validation sets were 0.790 (**Figure 5A**) and 0.780 (**Figure 6A**), respectively. In the external validation cohort, the model also achieved an AUC value of 0.780 (**Figure 7A**). The calibration curves in the three cohorts indicated that the nomogram has a good prediction ability (**Figures 5B**, **6B**, **7B**), with the predicted probability being highly consistent with the actual observed probability. In addition, the DCA curve confirmed the clinical utility of the nomogram (**Figures 5C**, **6C** **,** **7C**).

**Table 4:** Univariate and multivariable Logistic analysis of BCS benefit for young breast cancer patients.

	Univariate analysis			Multivariate analysis		
Variables	HR*	95%CI	*P-*value	HR*	95%CI	*P-*value
**Age**						
	1.004	(1.001, 1.007)	0.009	1.004	(1.002, 1.007)	0.002
**Race**						
Black	Reference					
Other	1.006	(0.972, 1.042)	0.724			
White	0.964	(0.939, 0.989)	0.005			
**Laterality**						
Left	Reference					
Right	1.011	(0.993, 1.03)	0.229			
**Marital**						
No	Reference			Reference		
Yes	0.959	(0.941, 0.977)	0.000	0.946	(0.93, 0.962)	0.000
**Grade**						
I	Reference			Reference		
II	0.868	(0.835, 0.902)	0.000	0.968	(0.935, 1.002)	0.067
III	0.889	(0.857, 0.923)	0.000	1.002	(0.968, 1.037)	0.900
IV	0.951	(0.882, 1.026)	0.196	1.018	(0.952, 1.089)	0.595
**Histology**						
IDC	Reference					
ILC	0.871	(0.806, 0.941)	0.001			
Other	1.023	(0.991, 1.056)	0.166			
**T stage**						
T1	Reference			Reference		
T2	0.926	(0.909, 0.944)	0.000	0.945	(0.928,0.962)	0.000
T3	0.730	(0.708, 0.753)	0.000	0.743	(0.722,0.765)	0.000
**N stage**						
N0	Reference			Reference		
N1	0.872	(0.854, 0.889)	0.000	0.884	(0.868, 0.900)	0.000
N2	0.808	(0.783, 0.834)	0.000	0.785	(0.762, 0.808)	0.000
N3	0.770	(0.739, 0.803)	0.000	0.775	(0.746, 0.805)	0.000
**Chemotherapy**						
No	Reference			Reference		
Yes	0.946	(0.924, 0.969)	0.000	0.928	(0.907, 0.950)	0.000
**Radiation**						
No	Reference			Reference		
Yes	1.382	(1.358, 1.406)	0.000	1.459	(1.435, 1.484)	0.000
**Subtype**						
HR-/HER2-	Reference			Reference		
HR-/HER2+	0.92	(0.861, 0.983)	0.014	0.972	(0.917, 1.031)	0.350
HR+/HER2-	0.992	(0.956, 1.030)	0.680	0.973	(0.940, 1.008)	0.126
HR+/HER2+	0.969	(0.925, 1.015)	0.185	0.966	(0.927, 1.008)	0.109
Not 2010+	1.152	(1.114, 1.191)	0.000	1.127	(1.093, 1.162)	0.000

BCS, Breast conserving surgery; IDC, Invasive ductal carcinoma; ILC, Invasive lobular carcinoma; HR, Hormone receptor; HER2, Human epidermal growth factor receptor 2; HR*, hazard ratio; CI, confidence interval.

**Figure 8:**
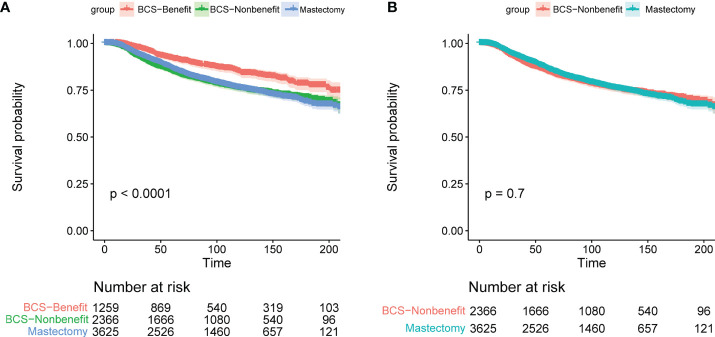
The KM survival analysis of patients in the BCS-Benefit group, BCS-Nonbenefit group, and mastectomy group.

Finally, we verified the use of the model in the PSM cohort. Based on the risk score in the nomogram, 1,259 patients were classified in the BCS-Benefit group, and 2,366 patients were classified in the BCS-Nonbenefit group. The KM survival curves were generated to observe the difference in survival benefits between groups (**Figure 8**). The results showed that the survival advantage of patients in the BCS-Benefit group was higher than that in the BCS-Nonbenefit or mastectomy ones (*P <*0.001). Moreover, there was no significant difference in OS between the BCS-non-benefit group and mastectomy one (*P* =0.700). These results indicated that not all young breast cancer patients benefit from BCS, and some have an equal benefit to a mastectomy.

## Discussion

In recent years, the incidence of breast cancer in women under the age of 40 and even 30 has continued to increase, while the prognosis for them is poor. In addition, there are more challenges and demands in the treatment of young breast cancer patients, as well as more socioeconomic implications. There is a lack of reliable evidence for the treatment decisions due to the small proportion of young breast cancer patients in clinical trials (11, 24). Therefore, it is necessary to determine the best way to manage the treatment of young breast cancer patients.

Sun et al. compared prognosis between BCT and mastectomy for early breast cancer in young patients under 40 years old, they found that there was no significant survival difference for 18-35 years old group (25). Quan et al. draw a similar conclusion (26). But two retrospective studies found that BCS could significantly improved prognosis in young breast cancer patients under the age of 40 (27, 28).However, none of the previous studies above have comprehensively establish a model to screen young breast cancer patients who are suitable for BCS. Our study is the first to quantify the benefit of BCS in young breast cancer patients by Logistic regression and to construct a nomogram. In our analysis, the cohort was divided into BCS group and mastectomy group. PSM was performed to eliminate demographic or pathological baseline imbalances between the two groups. We next observed the clinicopathological features between the two groups and identified BCS as an independent factor for OS and BCSS in young patients by univariate and multivariate Cox regression analyses. Finally, we screened out the factors affecting the benefit of BCS by univariate and multivariate Logistic regression and constructed a nomogram. The nomogram was validated that its predictive performance was favorable both in internal and external cohort. Our research showed that receiving BCS could improve the OS and BCSS of young patients, but not all of them benefited from it.

We found that most young patients should receive BCS and benefit from it. This conclusion is consistent with the recommendations of many breast cancer conference guidelines. The St. Gallen Consensus Group in 2013 stated that young age is not an absolute contraindication to BCS (29). The European Association of Breast Cancer Specialists (EUSOMA) working group suggested that BCT is the first choice for suitable young breast cancer patients (30). Moreover, the first International Consensus Conference on Breast Cancer in Young Women proposed the same recommendation (31). For young breast cancer patients, it is emphasized that there be a balance between tumor treatment efficacy, postoperative aesthetics, and long-term complications to protect their physical and mental health. In addition, young patients have a high risk of recurrence after BCS, and follow-up management should be strengthened.

Our analysis indicated that among patients under 35 years old, those who are older age, have lower T and N stage, radiation, as well as no chemotherapy, were associated with a benefit from BCS. Studies have shown that age is an independent risk factor for tumor recurrence after BCS (7). According to our nomogram, in patients <= 35 years old, younger age was associated with less benefit after receiving BCS. In addition, the key to BCS in clinical practice is to ensure that there is no residual tumor at the resection margin when removing the tumor. Therefore, BCS can be performed on patients with T1-3 stage who have an appropriate breast volume and a ratio of tumor to breast volume. Plastic repair techniques for tumors may improve breast shape and symmetry after BCS in young breast cancer patients. For N stage, preoperative confirmation of lymph node metastasis is not an absolute contraindication to BCS, and even for some N1 patients, BCS and postoperative radiotherapy can avoid further axillary lymph node dissection (32). However, patients with N1-3 breast cancer have higher local and regional recurrence risks compared with node-negative patients after BCS (33). Therefore, how to select T and N stages that are more suitable for BCS is crucial. Our model confirms that patients with lower T and N stages are more likely to benefit from BCS, which also provides a reference for clinicians to make decisions. In terms of systemic treatment, BCS followed by radiation is a widely accepted standard approach that allows for organ preservation in most early-stage breast cancers (8, 14). Our study also confirms that patients with postoperative radiation are more likely to benefit from BCS.

The greater benefit of BCS without chemotherapy than with chemotherapy is seen in Logistic regression, and the following aspects should be considered. The cohort study in 127 hospitals in the UK (POSH) and the breast cancer study in young women in Europe (HOHO) showed that young breast cancer patients had a higher proportion of HR+ tumor compared with older women (34, 35). Similar results were found in our findings, with the highest proportion of HR+HER2-type in our study cohort. ESO-ESMO 5th International Consensus Guidelines for Breast Cancer in Young Women (BCY5) confirmed that young breast cancer patients with luminal-like tumors have poorer outcome (2), which may explained by different tumor or host biological behavior, less chemotherapy-induced amenorrhea, poor endocrine therapy response, and poor adherence to adjuvant endocrine therapy etc. According to our analysis, beneficial was diminished after BCS for those received chemotherapy, which was generally associated with poor tumor features of patients received chemotherapy rather than treatment failure. However, considering that our study was a large retrospective study and the under-representation of young breast cancer patients, the results still need to be treated with caution, and more prospective studies are needed to be further verified in the future.

Despite our model having a promising predictive value in identifying appropriate candidates for BCS among young women with breast cancer, several limitations remain. First, some information is missing from the SEER database, such as BRCA1/2 mutation, Ki67, HER2 status before 2010, and tumor progression, which may affect the performance of the model. Second, the impact of systemic therapy on prognosis cannot be analyzed comprehensively, such as endocrine therapy, targeted therapy, and immune therapy. Third, PSM requires a large sample size to achieve high-quality matching, and may lose more data and cause the remaining samples to be unrepresentative. Finally, this is a retrospective study, which may have selection bias, and our findings need to be supplemented and validated with prospective studies.

In conclusion, our findings suggest that BCS can bring better OS and BCSS than mastectomy for young breast cancer patients, but not all benefit from it. Herein we constructed a model for young breast cancer patients (≤35 years old) which could identify appropriate candidates who may benefit from BCS. For patients assigned to the BCS-Nonbenefit group, their OS did not differ from those who received a mastectomy. These findings could provide a reference for clinicians in therapy decisions.

## Data availability statement

The dataset for this study can be found in the SEER database [https://seer.cancer.gov/]. The original contributions presented in the study are included in the article/**Supplementary Material**. Further inquiries can be directed to the corresponding authors.

## Author contributions

SP: Conceptualization, Methodology, Validation. Writing - Original Draft. SS, HC: Formal analysis, Investigation. CZ, HZ: Software, Visualization. KW: Data Curation, Resources. JH: Writing- Reviewing and Editing, Supervision. JZ: Conceptualization, Writing - Review and Editing. All authors read and approved the final manuscript. All authors contributed to the article and approved the submitted version.

## Acknowledgments

The authors thank AiMi Academic Services (www.aimieditor.com) for the English language editing and review services.

## Conflict of interest

The authors declare that the research was conducted in the absence of any commercial or financial relationships that could be construed as a potential conflict of interest.

## Publisher’s note

All claims expressed in this article are solely those of the authors and do not necessarily represent those of their affiliated organizations, or those of the publisher, the editors and the reviewers. Any product that may be evaluated in this article, or claim that may be made by its manufacturer, is not guaranteed or endorsed by the publisher.

## References

[1] Paluch-Shimon S Cardoso F Partridge AH Abulkhair O Azim HA Jr. Bianchi-Micheli G . ESO-ESMO 4th international consensus guidelines for breast cancer in young women (BCY4). Ann Oncol (2020) 31(6):674–96. doi: 10.1016/j.annonc.2020.03.284 32199930

[2] Paluch-Shimon S Cardoso F Partridge AH Abulkhair O Azim HA Bianchi-Micheli G . ESO-ESMO fifth international consensus guidelines for breast cancer in young women (BCY5). Ann Oncol (2022) S0923-7534(22):01858-0. doi: 10.1016/j.annonc.2022.07.007 35934170

[3] Braunstein LZ Taghian AG Niemierko A Salama L Capuco A Bellon JR . Breast-cancer subtype, age, and lymph node status as predictors of local recurrence following breast-conserving therapy. Breast Cancer Res Treat (2017) 161(1):173–9. doi: 10.1007/s10549-016-4031-5 27807809

[4] Han W Kang SY Korean Breast Cancer S . Relationship between age at diagnosis and outcome of premenopausal breast cancer: age less than 35 years is a reasonable cut-off for defining young age-onset breast cancer. Breast Cancer Res Treat (2010) 119(1):193–200. doi: 10.1007/s10549-009-0388-z 19350387

[5] Voogd AC Nielsen M Peterse JL Blichert-Toft M Bartelink H Overgaard M . Differences in risk factors for local and distant recurrence after breast-conserving therapy or mastectomy for stage I and II breast cancer: pooled results of two large European randomized trials. J Clin Oncol (2001) 19(6):1688–97. doi: 10.1200/JCO.2001.19.6.1688 11250998

[6] Chen HL Zhou MQ Tian W Meng KX He HF . Effect of age on breast cancer patient prognoses: A population-based study using the SEER 18 database. PloS One (2016) 11(10):e0165409. doi: 10.1371/journal.pone.0165409 27798652 PMC5087840

[7] Azim HA Jr. Partridge AH . Biology of breast cancer in young women. Breast Cancer Res (2014) 16(4):427. doi: 10.1186/s13058-014-0427-5 25436920 PMC4303229

[8] Fisher B Anderson S Bryant J Margolese RG Deutsch M Fisher ER . Twenty-year follow-up of a randomized trial comparing total mastectomy, lumpectomy, and lumpectomy plus irradiation for the treatment of invasive breast cancer. N Engl J Med (2002) 347(16):1233–41. doi: 10.1056/NEJMoa022152 12393820

[9] van Maaren MC de Munck L de Bock GH Jobsen JJ van Dalen T Linn SC . 10 year survival after breast-conserving surgery plus radiotherapy compared with mastectomy in early breast cancer in the Netherlands: a population-based study. Lancet Oncol (2016) 17(8):1158–70. doi: 10.1016/S1470-2045(16)30067-5 27344114

[10] Veronesi U Cascinelli N Mariani L Greco M Saccozzi R Luini A . Twenty-year follow-up of a randomized study comparing breast-conserving surgery with radical mastectomy for early breast cancer. N Engl J Med (2002) 347(16):1227–32. doi: 10.1056/NEJMoa020989 12393819

[11] Kroman N Holtveg H Wohlfahrt J Jensen MB Mouridsen HT Blichert-Toft M . Effect of breast-conserving therapy versus radical mastectomy on prognosis for young women with breast carcinoma. Cancer (2004) 100(4):688–93. doi: 10.1002/cncr.20022 14770422

[12] Litiere S Werutsky G Fentiman IS Rutgers E Christiaens MR Van Limbergen E . Breast conserving therapy versus mastectomy for stage I-II breast cancer: 20 year follow-up of the EORTC 10801 phase 3 randomised trial. Lancet Oncol (2012) 13(4):412–9. doi: 10.1016/S1470-2045(12)70042-6 22373563

[13] Vila J Gandini S Gentilini O . Overall survival according to type of surgery in young (</=40 years) early breast cancer patients: A systematic meta-analysis comparing breast-conserving surgery versus mastectomy. Breast (2015) 24(3):175–81. doi: 10.1016/j.breast.2015.02.002 25728282

[14] van Dongen JA Voogd AC Fentiman IS Legrand C Sylvester RJ Tong D . Long-term results of a randomized trial comparing breast-conserving therapy with mastectomy: European organization for research and treatment of cancer 10801 trial. J Natl Cancer Inst (2000) 92(14):1143–50. doi: 10.1093/jnci/92.14.1143 10904087

[15] Miles RC Gullerud RE Lohse CM Jakub JW Degnim AC Boughey JC . Local recurrence after breast-conserving surgery: multivariable analysis of risk factors and the impact of young age. Ann Surg Oncol (2012) 19(4):1153–9. doi: 10.1245/s10434-011-2084-6 21989658

[16] Botteri E Bagnardi V Rotmensz N Gentilini O Disalvatore D Bazolli B . Analysis of local and regional recurrences in breast cancer after conservative surgery. Ann Oncol (2010) 21(4):723–8. doi: 10.1093/annonc/mdp386 19833817

[17] Bantema-Joppe EJ de Munck L Visser O Willemse PH Langendijk JA Siesling S . Early-stage young breast cancer patients: impact of local treatment on survival. Int J Radiat Oncol Biol Phys (2011) 81(4):e553–9. doi: 10.1016/j.ijrobp.2011.02.060 21601378

[18] Hwang ES Lichtensztajn DY Gomez SL Fowble B Clarke CA . Survival after lumpectomy and mastectomy for early stage invasive breast cancer: the effect of age and hormone receptor status. Cancer (2013) 119(7):1402–11. doi: 10.1002/cncr.27795 PMC3604076 23359049

[19] Christiansen P Carstensen SL Ejlertsen B Kroman N Offersen B Bodilsen A . Breast conserving surgery versus mastectomy: overall and relative survival-a population based study by the Danish breast cancer cooperative group (DBCG). Acta Oncol (2018) 57(1):19–25. doi: 10.1080/0284186X.2017.1403042 29168674

[20] Agarwal S Pappas L Neumayer L Kokeny K Agarwal J . Effect of breast conservation therapy vs mastectomy on disease-specific survival for early-stage breast cancer. JAMA Surg (2014) 149(3):267–74. doi: 10.1001/jamasurg.2013.3049 24429935

[21] Desai RJ Franklin JM . Alternative approaches for confounding adjustment in observational studies using weighting based on the propensity score: a primer for practitioners. BMJ (2019) 367:l5657. doi: 10.1136/bmj.l5657 31645336

[22] Andrade C . Mean difference, standardized mean difference (SMD), and their use in meta-analysis: As simple as it gets. J Clin Psychiatry (2020) 81(5):20f13681. doi: 10.4088/JCP.20f13681 32965803

[23] Van Calster B Wynants L Verbeek JFM Verbakel JY Christodoulou E Vickers AJ . Reporting and interpreting decision curve analysis: A guide for investigators. Eur Urol (2018) 74(6):796–804. doi: 10.1016/j.eururo.2018.08.038 30241973 PMC6261531

[24] de Bock GH van der Hage JA Putter H Bonnema J Bartelink H van de Velde CJ . Isolated loco-regional recurrence of breast cancer is more common in young patients and following breast conserving therapy: long-term results of European organisation for research and treatment of cancer studies. Eur J Cancer (2006) 42(3):351–6. doi: 10.1016/j.ejca.2005.10.006 16314086

[25] Sun ZH Chen C Kuang XW Song JL Sun SR Wang WX . Breast surgery for young women with early-stage breast cancer: Mastectomy or breast-conserving therapy? Med (Baltimore) (2021) 100(18):e25880. doi: 10.1097/MD.0000000000025880 PMC8104198 33951002

[26] Quan ML Paszat LF Fernandes KA Sutradhar R McCready DR Rakovitch E . The effect of surgery type on survival and recurrence in very young women with breast cancer. J Surg Oncol (2017) 115(2):122–30. doi: 10.1002/jso.24489 28054348

[27] Lazow SP Riba L Alapati A James TA . Comparison of breast-conserving therapy vs mastectomy in women under age 40: National trends and potential survival implications. Breast J (2019) 25(4):578–84. doi: 10.1111/tbj.13293 31090168

[28] Yu P Tang H Zou Y Liu P Tian W Zhang K . Breast-conserving therapy versus mastectomy in young breast cancer patients concerning molecular subtypes: A SEER population-based study. Cancer Control (2020) 27(1):1073274820976667. doi: 10.1177/1073274820976667 33356518 PMC8480363

[29] Harbeck N Thomssen C Gnant M . St. gallen 2013: brief preliminary summary of the consensus discussion. Breast Care (Basel) (2013) 8(2):102–9. doi: 10.1159/000351193 PMC3683952 24000280

[30] Cardoso F Loibl S Pagani O Graziottin A Panizza P Martincich L . The European society of breast cancer specialists recommendations for the management of young women with breast cancer. Eur J Cancer (2012) 48(18):3355–77. doi: 10.1016/j.ejca.2012.10.004 23116682

[31] Partridge AH Pagani O Abulkhair O Aebi S Amant F Azim HA Jr. . First international consensus guidelines for breast cancer in young women (BCY1). Breast (2014) 23(3):209–20. doi: 10.1016/j.breast.2014.03.011 24767882

[32] Vane MLG Hunter-Squires J Kim S Smidt ML Giuliano AE . Women could avoid axillary lymph node dissection by choosing breast-conserving therapy instead of mastectomy. Ann Surg Oncol (2021) 28(5):2522–8. doi: 10.1245/s10434-021-09674-9 33586070

[33] Truong PT Jones SO Kader HA Wai ES Speers CH Alexander AS . Patients with t1 to t2 breast cancer with one to three positive nodes have higher local and regional recurrence risks compared with node-negative patients after breast-conserving surgery and whole-breast radiotherapy. Int J Radiat Oncol Biol Phys (2009) 73(2):357–64. doi: 10.1016/j.ijrobp.2008.04.034 18676091

[34] Copson ER Maishman TC Tapper WJ Cutress RI Greville-Heygate S Altman DG . Germline BRCA mutation and outcome in young-onset breast cancer (POSH): a prospective cohort study. Lancet Oncol (2018) 19(2):169–80. doi: 10.1016/S1470-2045(17)30891-4 PMC5805863 29337092

[35] Ruggeri M Pagan E Bagnardi V Bianco N Gallerani E Buser K . Fertility concerns, preservation strategies and quality of life in young women with breast cancer: Baseline results from an ongoing prospective cohort study in selected European centers. Breast (2019) 47:85–92. doi: 10.1016/j.breast.2019.07.001 31362134

