# Supplementary material for: A nomogram to identify appropriate candidates for breast-conserving surgery among young women with breast cancer: A large cohort study
Source: Front Oncol. 2022 Oct 21;12:1012689. doi: 10.3389/fonc.2022.1012689 (PMC9634247; doi:10.3389/fonc.2022.1012689)
Supplement: Supplementary file 1 [file Table_1.docx]

Supplementary table 1: Clinical and pathological characteristics for patients in the external validation cohort.

|  | **Total** | **Mastectomy** | **BCS** | ***P*-value** |
| --- | --- | --- | --- | --- |
| **Variables** |  | 87 (58.4%) | 62 (41.6%) |  |
| **Age** (Mean (SD)) | 31.4 (3.12) | 31.9 (2.57) | 30.7 (3.67) | 0.034 |
| **Marital** |  |  |  | 0.001 |
| No | 19 (12.8%) | 4 (4.60%) | 15 (24.2%) |  |
| Yes | 130 (87.2%) | 83 (95.4%) | 47 (75.8%) |  |
| **Laterality** |  |  |  | 0.074 |
| Left | 70 (47.0%) | 35 (40.2%) | 35 (56.5%) |  |
| Right | 79 (53.0%) | 52 (59.8%) | 27 (43.5%) |  |
| **Histology** |  |  |  | 0.105 |
| IDC | 81 (54.4%) | 53 (60.9%) | 28 (45.2%) |  |
| ILC | 2 (1.34%) | 1 (1.15%) | 1 (1.61%) |  |
| Other | 66 (44.3%) | 33 (37.9%) | 33 (53.2%) |  |
| **Stage_T** |  |  |  | 0.265 |
| T1 | 67 (45.0%) | 37 (42.5%) | 30 (48.4%) |  |
| T2 | 68 (45.6%) | 39 (44.8%) | 29 (46.8%) |  |
| T3 | 14 (9.40%) | 11 (12.6%) | 3 (4.84%) |  |
| **Stage_N** |  |  |  | 0.052 |
| N0 | 103 (69.1%) | 56 (64.4%) | 47 (75.8%) |  |
| N1 | 41 (27.5%) | 28 (32.2%) | 13 (21.0%) |  |
| N2 | 3 (2.01%) | 3 (3.45%) | 0 (0.00%) |  |
| N3 | 2 (1.34%) | 0 (0.00%) | 2 (3.23%) |  |
| **Radiation** |  |  |  | 0.024 |
| No | 58 (38.9%) | 41 (47.1%) | 17 (27.4%) |  |
| Yes | 91 (61.1%) | 46 (52.9%) | 45 (72.6%) |  |
| **Chemotherapy** |  |  |  | 0.968 |
| No | 37 (24.8%) | 21 (24.1%) | 16 (25.8%) |  |
| Yes | 112 (75.2%) | 66 (75.9%) | 46 (74.2%) |  |

Notes: BCS: Breast conserving surgery; IDC: Invasive ductal carcinoma; ILC: Invasive lobular carcinoma.

Supplementary Table 2. Univariate and multivariable analysis of breast cancer-specific survival (BCSS) predictors in breast cancer patients after PSM.

|  | **Univariate analysis** | | | **Multivariate analysis** | | |
| --- | --- | --- | --- | --- | --- | --- |
| **Variables** | **HR*** | **95%CI** | ***P-*value** | **HR*** | **95%CI** | ***P-*value** |
| **Age** | 0.980 | (0.962, 0.998) | 0.027 | 0.979 | (0.962,0.997) | 0.026 |
| **Race** |  |  |  |  |  |  |
| Black | Reference |  |  | Reference |  |  |
| Other | 0.587 | (0.474,0.728) | 0.000 | 0.709 | (0.571,0.881) | 0.002 |
| White | 0.643 | (0.558,0.739) | 0.000 | 0.704 | (0.611,0.811) | 0.000 |
| **Laterality** |  |  |  |  |  |  |
| Left | Reference |  |  |  |  |  |
| Right | 1.044 | (0.932,1.17) | 0.458 |  |  |  |
| **Marital** |  |  |  |  |  |  |
| No | Reference |  |  |  |  |  |
| Yes | 0.915 | (0.815,1.027) | 0.132 |  |  |  |
| **Grade** |  |  |  |  |  |  |
| I | Reference |  |  | Reference |  |  |
| II | 2.312 | (1.547,3.457) | 0.000 | 1.864 | (1.245,2.791) | 0.002 |
| III | 3.407 | (2.306,5.033) | 0.000 | 2.377 | (1.602,3.527) | 0.000 |
| IV | 3.088 | (1.818,5.243) | 0.000 | 2.433 | (1.429,4.142) | 0.001 |
| **Histology** |  |  |  |  |  |  |
| IDC | Reference |  |  |  |  |  |
| ILC | 1.062 | (0.658,1.715) | 0.805 |  |  |  |
| Other | 0.777 | (0.626,0.966) | 0.023 |  |  |  |
| **T stage** |  |  |  |  |  |  |
| T1 | Reference |  |  | Reference |  |  |
| T2 | 1.772 | (1.565,2.008) | 0.000 | 1.385 | (1.217,1.576) | 0.000 |
| T3 | 2.486 | (2.017,3.063) | 0.000 | 1.851 | (1.493,2.295) | 0.000 |
| **N stage** |  |  |  |  |  |  |
| N0 | Reference |  |  | Reference |  |  |
| N1 | 1.849 | (1.608,2.126) | 0.000 | 1.776 | (1.532,2.059) | 0.000 |
| N2 | 3.113 | (2.621,3.698) | 0.000 | 2.818 | (2.344,3.388) | 0.000 |
| N3 | 5.439 | (4.462,6.629) | 0.000 | 4.657 | (3.776,5.744) | 0.000 |
| **Chemotherapy** |  |  |  |  |  |  |
| No | Reference |  |  | Reference |  |  |
| Yes | 1.495 | (1.266,1.765) | 0.000 | 0.979 | (0.816,1.175) | 0.821 |
| **Radiation** |  |  |  |  |  |  |
| No | Reference |  |  | Reference |  |  |
| Yes | 1.479 | (1.313,1.666) | 0.000 | 1.001 | (0.874,1.148) | 0.984 |
| **Surgery** |  |  |  |  |  |  |
| BCS | Reference |  |  | Reference |  |  |
| Mastectomy | 1.137 | (1.015,1.274) | 0.027 | 1.126 | (1.004,1.263) | 0.042 |
| **Subtype** |  |  |  |  |  |  |
| HR-/HER2- | Reference |  |  | Reference |  |  |
| HR-/HER2+ | 0.453 | (0.226,0.908) | 0.026 | 0.473 | (0.236,0.95) | 0.035 |
| HR+/HER2- | 0.42 | (0.299,0.589) | 0.000 | 0.477 | (0.338,0.671) | 0.000 |
| HR+/HER2+ | 0.254 | (0.145,0.444) | 0.000 | 0.273 | (0.155,0.479) | 0.000 |
| Not 2010+ | 0.719 | (0.558,0.927) | 0.011 | 0.726 | (0.561,0.94) | 0.015 |

Notes: BCS: Breast conserving surgery; IDC: Invasive ductal carcinoma; ILC: Invasive lobular carcinoma; HR: Hormone receptor; HER2: Human epidermal growth factor receptor 2; HR*: hazard ratio; CI: confidence interval.

Supplementary Table 3. The risk point of each variable according to nomogram**.**

|  | **Variables** | **Points** |
| --- | --- | --- |
| **Age** | 8 | 0 |
|  | 10 | 2 |
|  | 12 | 4 |
|  | 14 | 6 |
|  | 16 | 8 |
|  | 18 | 10 |
|  | 20 | 12 |
|  | 22 | 14 |
|  | 24 | 17 |
|  | 26 | 19 |
|  | 28 | 21 |
|  | 30 | 23 |
|  | 32 | 25 |
|  | 34 | 27 |
|  | 35 | 29 |
| **Marital** | No | 13 |
|  | Yes | 0 |
| **T stage** | T1 | 94 |
|  | T2 | 81 |
|  | T3 | 0 |
| **N stage** | N0 | 75 |
|  | N1 | 38 |
|  | N2 | 4 |
|  | N3 | 0 |
| **Radiation** | No | 0 |
|  | Yes | 100 |
| **Chemotherapy** | No | 20 |
|  | Yes | 0 |

Supplementary Table 4. The total point and benefit possibility from breast conserving surgery (BCS) according to nomogram**.**

| **Total point** | **Benefit possibility** |
| --- | --- |
| 18 | 0.01 |
| 100 | 0.05 |
| 137 | 0.1 |
| 178 | 0.2 |
| 204 | 0.3 |
| 226 | 0.4 |
| 246 | 0.5 |
| 267 | 0.6 |
| 288 | 0.7 |
| 315 | 0.8 |
